# Pancreaticobiliary Fistula Caused by Intraductal Papillary Mucinous Adenoma Requiring Pancreaticoduodenectomy

**DOI:** 10.70352/scrj.cr.25-0779

**Published:** 2026-03-20

**Authors:** Mina Nagao, Hironobu Suto, Hiroyuki Matsukawa, Junichi Fujiwara, Seiko Kagawa, Takuro Fuke, Yoshio Shimizu, Arata Nishigaki, Yasuhisa Ando, Minoru Oshima, Keiichi Okano

**Affiliations:** 1 Department of Gastroenterological Surgery, Kagawa University, Kita, Kagawa, Japan; 2 Department of Diagnostic Pathology, Kagawa University, Kita, Kagawa, Japan

**Keywords:** intraductal papillary mucinous neoplasm, pancreaticobiliary fistula, obstructive jaundice, surgery

## Abstract

**INTRODUCTION:**

Fistula formation from the intraductal papillary mucinous neoplasm (IPMN) into neighboring organs is rare. We describe a case of pancreaticobiliary fistula with obstructive jaundice caused by an intraductal papillary mucinous adenoma (IPMA).

**CASE PRESENTATION:**

An 81-year-old man who was incidentally diagnosed with IPMN 11 years previously based on follow-up CT performed after nephrectomy for renal cell carcinoma. Gradual dilation of the main pancreatic duct was observed over time, confirming the high-risk stigmata for which surgery was recommended; however, the patient declined and was managed under surveillance. At a routine diabetes clinic visit, blood tests revealed inflammatory markers and hepatobiliary enzymes. Contrast-enhanced CT revealed a pancreatic head IPMN in extensive contact with the common bile duct with partial communication. Endoscopic nasobiliary drainage (ENBD) was performed, but resolution of jaundice proved difficult due to obstruction by mucin; therefore, we performed a subtotal stomach-preserving pancreaticoduodenectomy (SSPPD). Severe inflammation was observed around the pancreas and bile ducts at the surgical site. The postoperative course was favorable, and the patient was discharged on the POD 16. The histopathological diagnosis was intestinal-type IPMA.

**CONCLUSIONS:**

Most reported IPMN-related organ fistulas result from direct invasion by IPMC. By contrast, IPMA-related fistulation is exceedingly rare. We present an intestinal-type IPMA complicated by pancreaticobiliary fistula in which mucin defeated ENBD, necessitating SSPPD, and the patient had an uneventful recovery. This case shows that fistulation can occur even without IPMC and that early surgery should be considered in IPMN patients with pancreatobiliary fistula.

## Abbreviations

ENBD
: endoscopic nasobiliary drainage
IPMA
: intraductal papillary mucinous adenoma
IPMC
: intraductal papillary mucinous carcinoma
IPMN
: intraductal papillary mucinous neoplasm
PanIN
: pancreatic intraepithelial neoplasia
SSPPD
: subtotal stomach-preserving pancreaticoduodenectomy

## INTRODUCTION

Intraductal papillary mucinous neoplasm (IPMN) is a cystic tumor of the pancreas characterized by papillary proliferation of the mucinous epithelium within the pancreatic ducts. International consensus guidelines recommend resection in patients with “high-risk stigmata,” such as main duct dilation ≥10 mm, enhanced mural nodules, or obstructive jaundice.^1)^ Although the natural history of IPMN includes the risk of malignant transformation, fistula formation in adjacent organs is uncommon.^2)^ In addition, almost all fistula formation in adjacent organs cases are linked to intraductal papillary mucinous carcinoma (IPMC). Herein, we present an extremely rare case of an intraductal papillary mucinous adenoma (IPMA) with a pancreaticobiliary fistula that caused obstructive jaundice and cholangitis and required pancreaticoduodenectomy.

## CASE PRESENTATION

The patient was an 81-year-old man who was incidentally diagnosed with IPMN 11 years previously based on follow-up CT performed after nephrectomy for renal cell carcinoma. Gradual dilation of the main pancreatic duct was observed over time, confirming the high-risk stigmata for which surgery was recommended; however, the patient declined and was managed under surveillance. His medical history included the presence of hypertension and diabetes mellitus. He had a smoking history of 20 cigarettes per day for 60 years and did not consume alcohol.

**Fig. 1:**
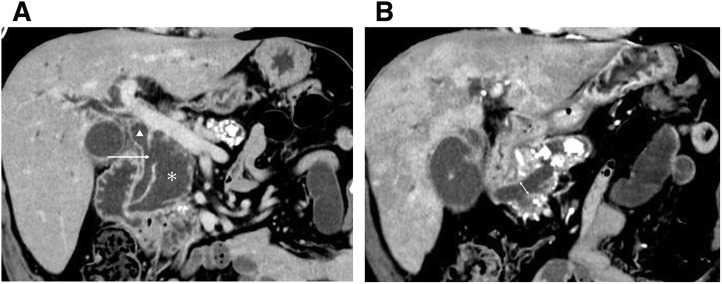
CT image. (**A**) Contrast-enhanced CT image showing communication between the IPMN and the common bile duct (*; IPMN, ▲; common bile duct, arrow; communication between IPMN and common bile duct). (**B**) Main pancreas duct enlarged 11 mm at pancreas head. Pancreatic stones are present throughout the pancreas (double-headed arrow; maximum diameter of main pancreas duct). IPMN, intraductal papillary mucinous neoplasm

**Fig. 2:**
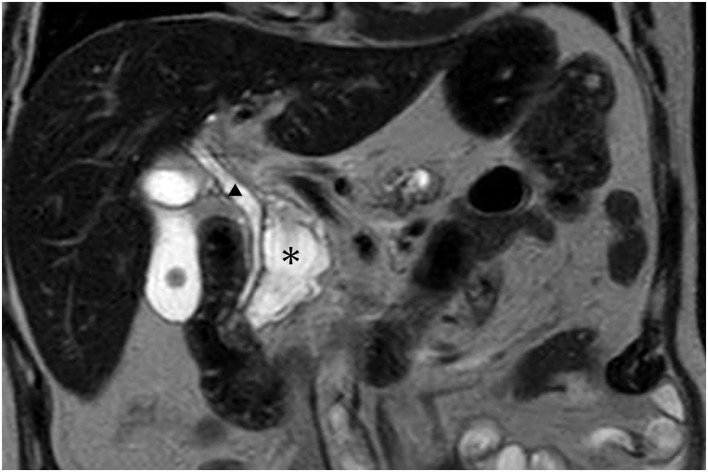
MRI image. MRI performed 1 month earlier showed no communication between the IPMN and the common bile duct (*; IPMN, ▲; common bile duct). IPMN, intraductal papillary mucinous neoplasm

At a routine diabetes clinic visit, laboratory tests showed elevated inflammatory markers and hepatobiliary enzymes (CRP 8.01 mg/dL, WBC 87 × 10^^2^, total bilirubin 14.1 mg/dL, direct bilirubin 9.5 mg/dL, AST [GOT] 50 U/L, ALT [GPT] 94 U/L, ALP 586 U/L, γ-GTP [GGT] 541 U/L). Contrast-enhanced CT revealed an IPMN approximately 35 mm in size in the pancreatic head without cyst wall thickening or intracystic nodules. The lesion broadly abutted and partially communicated with the common bile duct, with dilatation of both the intrahepatic and extrahepatic bile ducts (**Fig. 1A**). Numerous calcifications were present in the pancreatic parenchyma, and pancreatic stones were scattered within the main pancreatic duct (**Fig. 1B**). Increased fat stranding was observed in the hepatoduodenal ligament and around the pancreatic head. MRI imaging performed 1 month earlier showed no communication between the IPMN and the common bile duct (**Fig. 2**).

**Fig. 3:**
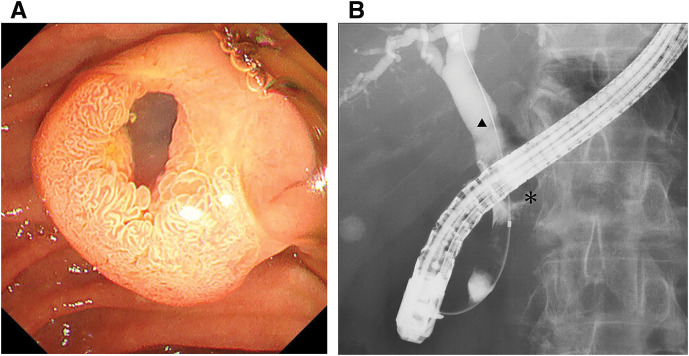
Endoscopic image. (**A**) ERCP revealed a patulous major duodenal papilla with mucin extrusion. (**B**) Cholangiography revealed communication between the IPMN and the common bile duct (*; IPMN, ▲; common bile duct). ERCP, endoscopic retrograde cholangiopancreatography; IPMN, intraductal papillary mucinous neoplasm

Based on these findings, the patient was admitted to the department of gastroenterology with a diagnosis of obstructive jaundice and acute cholangitis due to a pancreaticobiliary fistula arising from an IPMN. Endoscopic retrograde cholangiopancreatography revealed a patulous major duodenal papilla with mucin extrusion (**Fig. 3A**). Cholangiography revealed communication between the common bile duct and the IPMN (**Fig. 3B**). Endoscopic nasobiliary drainage (ENBD) was performed; however, the catheter was easily occluded by mucin, and effective biliary decompression remained difficult despite manual irrigation. Bile cytology from the ENBD was class II.

Because bile duct invasion by IPMC was suspected, surgery was considered more appropriate than additional drainage. Following informed consent for surgical management, the patient was referred to our department and a subtotal stomach-preserving pancreaticoduodenectomy with regional lymph node dissection was performed 28 days after ENBD. Preoperative laboratory tests showed near-resolution of inflammatory markers but persistent jaundice (CRP 0.26 mg/dL, WBC 63.7 × 10^2^, total bilirubin 5.9 mg/dL, direct bilirubin 3.9 mg/dL, AST [GOT] 38 U/L, ALT [GPT] 28 U/L, ALP 133 U/L, γ-GTP [GGT] 93 U/L). Intraoperatively, marked inflammation was observed around the pancreas and bile duct. Pancreatic transection was performed along the left border of the portal vein, and pancreatic stones in the remnant pancreas were retrieved to the extent feasible. The operative time was 8 h 55 min with an estimated blood loss of 907 mL, and no transfusion was required.

**Fig. 4:**
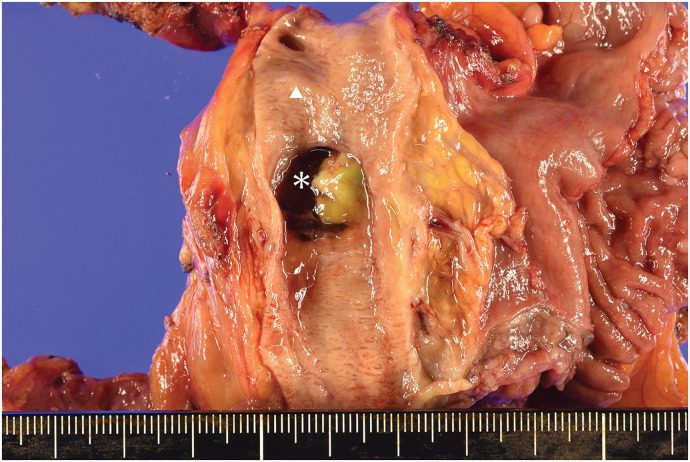
Resected specimen. Resected specimen showed that the cyst formed a fistula with the common bile duct, and the lumen was filled with mucin (*; IPMN, ▲; common bile duct). IPMN, intraductal papillary mucinous neoplasm

**Fig. 5:**
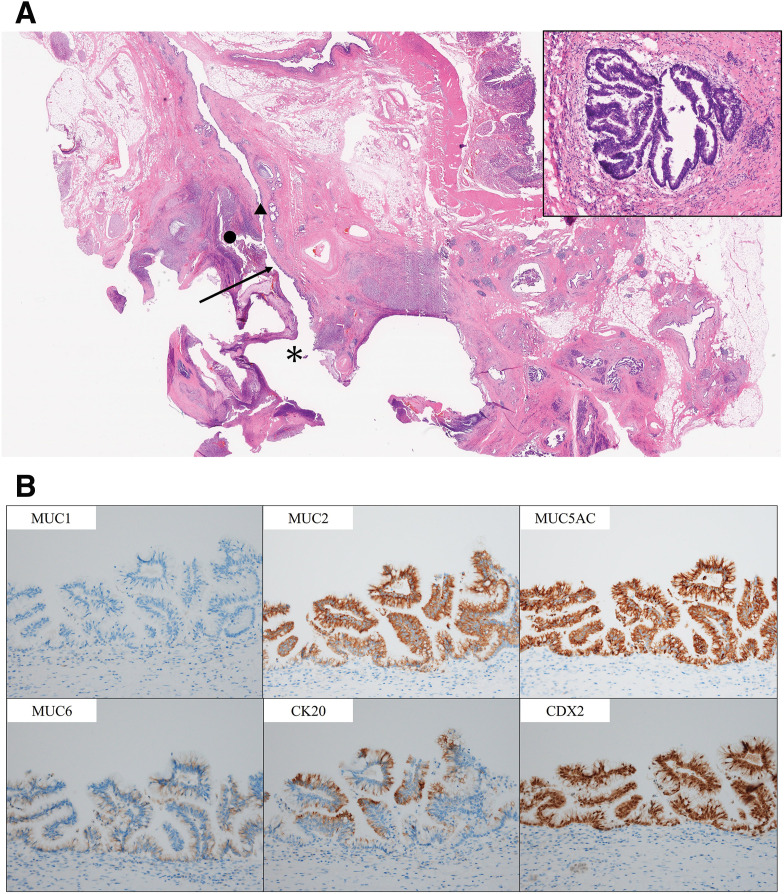
Pathological examination. (**A**) In the lesion, tall columnar tumor cells with pseudocircular nuclei and mucus proliferate in a papillary or tubular pattern. The IPMA has perforated into the bile duct and formed an abscess (*; IPMA, ▲; common bile duct, ●; abscess, arrow; fistula between IPMA and common bile duct). (**B**) Immunohistochemistry showed that the tumor cells were MUC1 (−), MUC2 (+), MUC5AC (+), MUC6 (−), CK20 (+), and CDX2 (+). IPMA, intraductal papillary mucinous adenoma

The resected specimen showed that the cyst had formed a fistula with the common bile duct and that the lumen was filled with mucin (**Fig. 4**). Histopathologically, the tumor measured 35 × 25 mm, with papillary proliferation of mucin-producing tumor cells within the pancreatic duct. Although prominent inflammatory cell infiltration was observed, no carcinoma was identified. No histological continuity of the tumor in the biliary epithelium was observed (**Fig. 5A**). On immunohistochemistry, the tumor cells were MUC1 (−), MUC2 (+), MUC5AC (+), MUC6 (-), CK20 (+), and CDX2 (+) (**Fig. 5B**). The final diagnosis was intestinal-type IPMA. No premalignant lesions, such as pancreatic intraepithelial neoplasia (PanIN), were identified at the pancreatic transection margin. Multiple pancreatic stones were observed, with histopathology confirming chromic pancreatitis. The postoperative course was uneventful, and the patient was discharged on POD 16.

## DISCUSSION

IPMN is a mucin-producing epithelial neoplasm of the pancreatic ductal system and a well-recognized precursor to invasive carcinoma. Contemporary international guidelines stratify risk by anatomic type (main-duct, branch-duct, or mixed) and by “high-risk stigmata” and “worrisome features,” providing algorithms for surveillance and resection.^1)^ High-risk stigmata, classically obstructive jaundice, an enhancing mural nodule ≥5 mm, or a main pancreatic duct diameter ≥10 mm, warrant resection in surgically fit patients. Reports of observation despite high-risk stigmata exist but are exceptional and highly selected, older and notably rarely symptomatic (no jaundice).^3,4)^ However, interval surgery is frequently required during the follow-up. In our case, the main pancreatic duct measured >10 mm, fulfilling high-risk stigmata, and despite a recommendation for surgery, the patient elected to undergo observation and was managed with surveillance.

Fistula formation from the IPMN into neighboring organs is rare but well-described, most commonly involving the duodenum, stomach, and bile duct.^5)^ Approximately, 97.1% of pancreaticobiliary fistula cases cause obstructive jaundice.^6)^ **Table 1** shows the literature summary of IPMN with pancreaticobiliary fistula. The proposed mechanisms include direct invasion by carcinoma and mechanical penetration driven by intraductal hypertension and proteolytic activity from copious mucin without histological invasion at the fistula edge.^7)^ In previously reported cases, mechanical perforation has been more common than invasion of cancer. As for treatment, endoscopic drainage is often hampered by recurrent occlusion from highly viscous mucin, and repeated exchanges and manual irrigation may provide only transient relief, as observed in this case. EUS-guided hepaticogastrostomy has been reported as a salvage or bridging option when transpapillary drainage fails; however, evidence remains limited.^8)^ Several reports in which surgery was not undertaken due to comorbidities or advanced age, endoscopic treatment was selected, and the clinical course remained poor without meaningful improvement.^9,10)^ In surgically fit patients with persistent jaundice/infection or ongoing risk owing to high-risk stigmata, pancreaticoduodenectomy represents a definitive treatment, with several reports noting favorable recovery.^11)^ Accordingly, endoscopic therapy has inherent limitations, and in our case the lack of improvement with ENBD, along with the potential for IPMC, the decision to undertake surgical treatment was appropriate.

**Table 1:** Literature summary of IPMN with pancreticobiliary fistula

Author	Year	Age/Sex	Proposed mechanism	Treatment	Pathological diagnosis	Prognosis (months)
Kurihara et al.^15)^	2000	74/M	Mechanical perforation	PD	IPMC	Dead (72)
Kurihara et al.^15)^	2000	87/M	Mechanical perforation	PD	IPMC	Alive (24)
Corguille et al.^16)^	2002	81/M	NA	Endoscopic treatment (stent)	NA	NA
Sano et al.^17)^	2003	70/M	Mechanical perforation	PD	IPMC	NA
Okada et al.^7)^	2008	67/M	Mechanical perforation	PD	IPMC	Alive (7)
Nagano et al.^18)^	2009	71/M	Invasion of cancer	PD	IPMC	NA
Bong et al.^19)^	2011	36/M	Invasion of cancer	PD	IPMC	Alive (6)
Sung et al.^9)^	2011	69/M	Mechanical perforation	Endoscopic treatment (EST)	NA	Alive (10)
Goto et al.^20)^	2012	75/M	Mechanical perforation	Endoscopic treatment (stent)	NA	Alive (6)
Mihara et al.^21)^	2015	77/M	Mechanical perforation	PD	IPMC	NA
Koizumi et al.^10)^	2016	84/F	NA	Endoscopic treatment (stent)	NA	Dead (0)
Koizumi et al.^10)^	2016	87/F	NA	Endoscopic treatment (ENBD)	NA	Dead (NA)
Koizumi et al.^10)^	2016	90/M	NA	Endoscopic treatment (stent)	NA	NA
Komo et al.^11)^	2018	79/M	Invasion of cancer	PD	IPMC	Alive (9)
Ren et al.^22)^	2019	52/M	Mechanical perforation	PD	IPMN (intermediate-grade dysplasia) IPNB (high-grade dysplasia)	Alive (52)
Khneizer et al.^23)^	2019	57/M	NA	Endoscopic treatment (stent)	IPMN (intermediate-grade dysplasia)	NA
Okamoto et al.^24)^	2019	87/M	NA	Endoscopic treatment (septotomy)	NA	NA
Kumar et al.^25)^	2021	60/F	NA	PTBD	NA	Dead (NA)
Mie et al.^8)^	2021	87/M	NA	Endoscopic treatment (EUS-HGS)	NA	NA
Patel et al.^26)^	2023	89/F	Mechanical perforation	Endoscopic treatment (stent)	IPMN (high-grade dysplasia)	Alive (9)
Goncalves et al.^27)^	2024	81/M	NA	PD	IPMN (high-grade dysplasia)	NA
Itagaki et al.^6)^	2025	70/F	Mechanical perforation	PD	IPMC	NA
Our case	2025	81/M	Mechanical perforation	PD	IPMA	Alive (5)

ENBD, endoscopic nasobiliary drainage; EST, endoscopic sphincterotomy; EUS-HGS, endoscopic ultrasound-guided hepaticogastrostomy; IPMA, intraductal papillary mucinous adenoma; IPMC, intraductal papillary mucinous carcinoma; IPMN, intraductal papillary mucinous neoplasm; NA, not applicable; PD, pancreaticoduodenectomy

Fistulation of IPMN into other organs has been described in the literature, but most cases are linked to IPMC. By contrast, fistula formation arising from IPMA has been reported only in a few isolated case reports. Jausset et al.^12)^ reported pancreaticogastric and pandreaticoduodenal fistulas arising from benign IPMN. As shown in **Table 1**, no prior reports have reported pancreaticobiliary fistula by IPMA, indicating that our case is exceedingly rare. Fistulation of IPMA cases typically ascribed to mucin hypersecretion with consequent intraductal hypertension, underscoring the fact that fistulation does not necessarily equate to an invasive pathology.^13)^ Mucin quantity/viscosity correlates more with histologic subtype than with grade. Intestinal-type IPMN, typically MUC1(−), MUC2(+), CDX2(+), is characteristically mucin-hypersecretory, predisposing to intraductal hypertension and, in rare instances, fistulation; pancreatobiliary-type tends to show MUC1 expression and is more often associated with invasive tubular components and worse outcomes. While invasive transformation frequently coincides with faster growth and accumulation of high-risk features, mucin overproduction is not exclusive to carcinoma and can be prominent in noninvasive intestinal-type lesions. The immunophenotype in our case (MUC1 (−), MUC2 (+), MUC5AC (+), MUC6 (−), CK20 (+), and CDX2 (+)) aligns with intestinal-type IPMA and explains the heavy mucin burden despite the absence of carcinoma. While chronic pancreatitis reported to promote fistula formation in IPMN,^14)^ the mechanism is unknown, and it remains uncertain whether it contributed to the pancreaticobiliary fistula in our case.

## CONCLUSIONS

Although most IPMN-related organ fistulas stem from direct invasion by IPMC, IPMA-related fistulation is exceptionally uncommon. In our intestinal-type IPMA, viscous mucin defeated ENBD, prompting SSPPD. These findings indicate that fistulation may occur in the benign IPMN and support early surgical intervention in IPMN with a pancreatobiliary fistula.
